# Rationale for Utilization of Hydrogel Rectal Spacers in Dose Escalated SBRT for the Treatment of Unfavorable Risk Prostate Cancer

**DOI:** 10.3389/fonc.2022.860848

**Published:** 2022-03-31

**Authors:** Michael C. Repka, Michael Creswell, Jonathan W. Lischalk, Michael Carrasquilla, Matthew Forsthoefel, Jacqueline Lee, Siyuan Lei, Nima Aghdam, Shaan Kataria, Olusola Obayomi-Davies, Brian T. Collins, Simeng Suy, Ryan A. Hankins, Sean P. Collins

**Affiliations:** ^1^ Department of Radiation Oncology, University of North Carolina School of Medicine, Chapel Hill, NC, United States; ^2^ Georgetown University School of Medicine, Washington, DC, United States; ^3^ Department of Radiation Oncology at New York University (NYU) Long Island School of Medicine, Perlmutter Cancer Center at NYCyberKnife, New York, NY, United States; ^4^ Department of Radiation Medicine, MedStar Georgetown University Hospital, Washington, DC, United States; ^5^ Department of Radiation Oncology, Radiotherapy Centers of Kentuckiana, Louisville, KY, United States; ^6^ Department of Radiation Oncology, Beth Israel Deaconess Medical Center, Boston, MA, United States; ^7^ Department of Radiation Oncology, Arlington & Reston Radiation Oncology, Arlington, VA, United States; ^8^ Department of Radiation Oncology, Wellstar Kennestone Hospital, Marietta, GA, United States; ^9^ Department of Urology, MedStar Georgetown University Hospital, Washington, DC, United States

**Keywords:** prostate, hydrogel, rectal spacer, radiotherapy, radiation therapy, SBRT

## Abstract

In this review we outline the current evidence for the use of hydrogel rectal spacers in the treatment paradigm for prostate cancer with external beam radiation therapy. We review their development, summarize clinical evidence, risk of adverse events, best practices for placement, treatment planning considerations and finally we outline a framework and rationale for the utilization of rectal spacers when treating unfavorable risk prostate cancer with dose escalated Stereotactic Body Radiation Therapy (SBRT).

## Introduction

Prostate cancer is the most common cancer diagnosed in male patients in the United States with an estimated 248,530 cases in 2021 (1). Greater than 80% of these patients present with either localized or regional disease, and the vast majority of this subset may be eligible for curative treatment with radiotherapy. In general, biochemical disease free-survival and long-term overall survival rates are excellent in patients treated with definitive radiotherapy, even for those patients with high-risk or node-positive disease (2). However, although acute toxicity in these patients tends to be mild and self-limiting, some patients may experience late effects of radiotherapy that can be morbid and difficult to manage (3). In particular, long-term randomized quality of life (QoL) data suggest that while urinary and sexual function are at least comparable if not better than radical prostatectomy, there are higher degrees of bowel bother and rectal bleeding with definitive radiotherapy (4, 5). In rare cases, life threatening late events including fistula formation and soft tissue necrosis have been reported following dose-escalated radiotherapy (6). Multiple strategies to mitigate long-term rectal toxicity have been employed including sophisticated radiation techniques such as intensity modulated radiotherapy (IMRT), proton beam therapy (PBT), and physical devices such as rectal balloons and implanted materials to physically separate the posterior aspect of the prostate from the anterior rectal wall (7–9). In this article, we review the development, data, and rationale for utilization of hydrogel rectal spacers in prostate SBRT dose escalation for unfavorable risk prostate cancer.

## Hydrogel Rectal Spacer Background and Development

Regardless of treatment site and modality, radiation dose is often only limited by the dose constraints of the surrounding organs at risk. On account of the intimate association between the posterior prostate and the anterior wall of the rectum, significant interest has arisen in developing a means of physical separation between the two organs to reduce radiation-induced rectal toxicity. The posterior prostate and seminal vesicles are separated from the rectum by a fibromuscular structure known as the rectoprostatic (Denonvilliers’) fascia (10). During radical prostatectomy, the tissue plane posterior to this fascia and anterior to the muscular wall of the rectum is dissected and exploitation of this potential space has proven attractive for creating artificial geometric separation between the prostate and rectum for patient’s undergoing non-operative treatments such as cryoablation (11).

Multiple different space-creating solutions have been developed over the past 10-15 years, including an implanted bio-absorbable balloon, hyaluronic acid, human collagen, and polyethylene glycol (PEG) based hydrogel (12–14). Of these various methods, the hydrogel spacer is the most widely used and has the largest wealth of supporting clinical data. In fact, extensive experience with PEG based hydrogels in humans existed prior to the development of the rectal spacer – they have been used as sealants following vascular puncture, dural repair, and pleural decortication (15–17). After placement, the hydrogel remains solid for approximately 3 months before it begins to resorb, which typically occurs by 6 months. Complete resorption in 100% of patients is seen 9 months post-placement (18).

The most widely available rectal spacer formulation, marketed as SpaceOAR™, was initially developed by a start-up company called Augmenix and received Food and Drug Administration (FDA) approval in 2015 (19). Augmenix was subsequently purchased by Boston Scientific in 2018 (20). SpaceOAR Vue™ is a newer, similar PEG hydrogel with approximately 1% iodine, allowing improved visualization on CT-based imaging and accurate spacer delineation in patients with a contraindication to MRI (21).

## Hydrogel Rectal Spacer in Practice and Clinical Data

A single, prospective, multi-center phase III randomized trial represents the highest level of evidence in support of rectal spacer application (18, 22–24). In this study, 222 patients with clinical T1 or T2 prostate cancer were randomized to dose escalated image-guided IMRT with or without hydrogel rectal spacer placement prior to treatment. All patients received intraprostatic fiducial markers for image-guided radiotherapy (IGRT) and were treated to a total dose of 79.2 Gy in 1.8 Gy daily fractions. Pelvic lymph nodes were not included in the clinical target volume (CTV), and seminal vesicles were included at the treating physician’s discretion. Statistically significant dosimetric improvements were identified in the rectal volume receiving at least 50 Gy, 60 Gy, 70 Gy, and 80 Gy. With a median follow-up of three years, patients who received a rectal spacer experienced a significantly lower incidence of grade 1 and grade 2 rectal toxicity, as well as grade 1 urinary toxicity (22). Patient reported QoL outcomes were also better in those patients with a rectal spacer, and secondary analysis suggested improvements in long-term sexual function as well – hypothetically due to lower dose to other OARs such as the penile bulb made feasible by easier attainment of rectal constraints (23).

**Figure 1:**
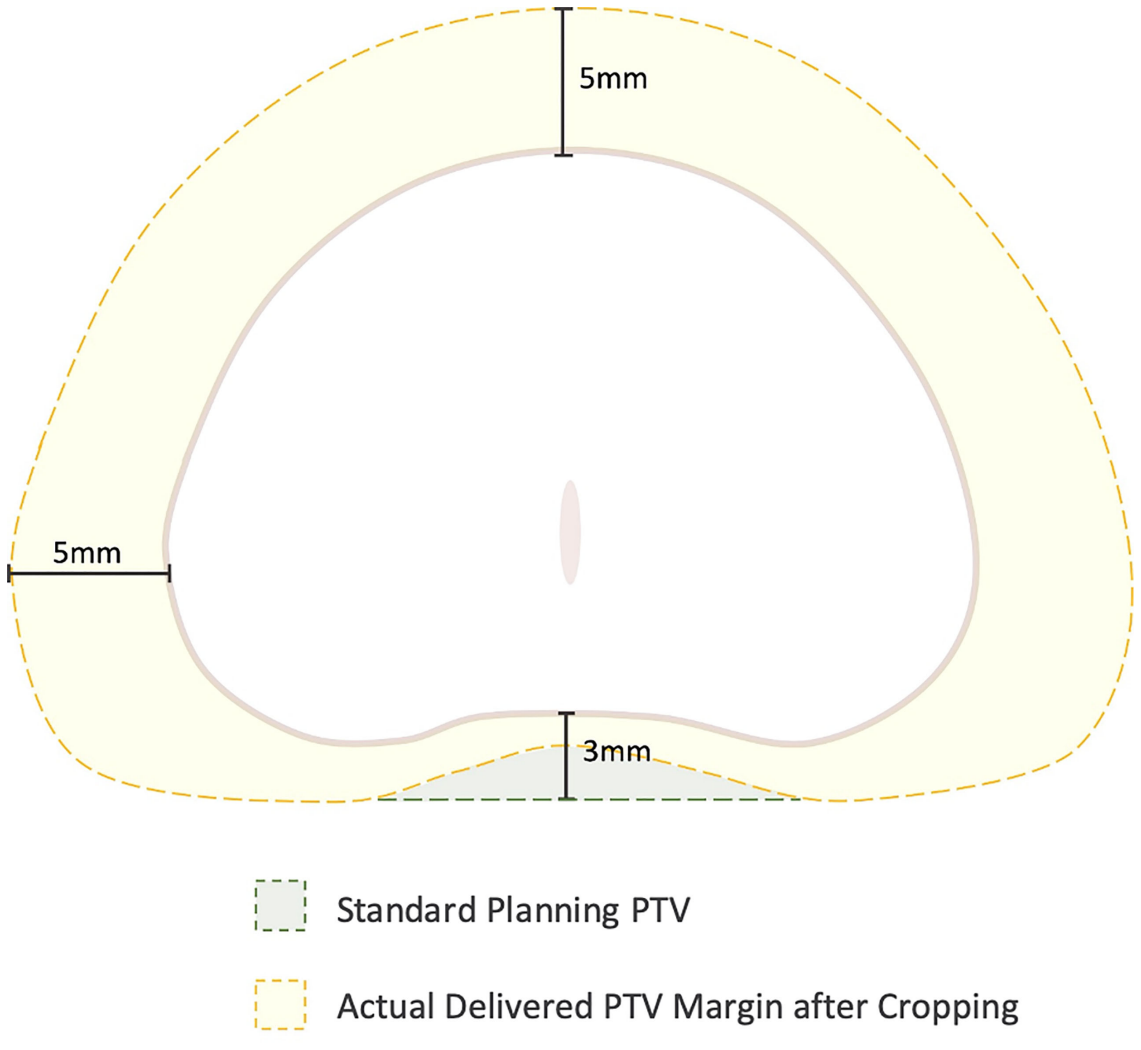
Cropping of the Planning Target Volume (PTV) secondary to stringent rectal dose constraints. In general, the PTV is formed by expanding the prostate volume 3 mm posteriorly and 5 mm in all other dimensions. However, to achieve rectal dose constraints, the posterior margin is commonly cropped out of the rectum leading to a “true” posterior margin on such plans closer to 1-2 mm.

Multiple other non-randomized studies have been performed which demonstrate the dosimetric and clinical benefits to hydrogel rectal spacer placement. Beyond improving clinical outcomes for patients treated with conventionally fractionated radiotherapy (e.g., 79.2 Gy in 44 fractions) or moderately hypofractionated treatment (e.g., 60 Gy in 20 fractions), there is considerable interest in utilizing the technology to allow for greater dose escalation, particularly in patients treated with SBRT. Although SBRT for patients with low- and intermediate-risk disease typically experience low rates of late toxicity with typical dosing (35 – 36.25 Gy in 5 fractions) (25), substantial rectal toxicity has been reported in patients treated with more aggressive regimens. For instance, in one phase II dose escalation study from the University of Texas – Southwestern, patients received escalating doses up to 50 Gy in 5 fractions, with relatively high rates of severe toxicity in this cohort (e.g. rectourethral fistula) including 5 patients who required colostomy (26, 27). Interestingly, 5 year biochemical disease control and distant metastasis free survival were 98.6% and 100%, respectively suggesting a benefit to dose escalation (28). Furthermore, the excellent long-term toxicity outcomes reported in patients treated with more typical SBRT dose regimens are achieved by maintaining strict rectal dose constraints, often at the cost of tight posterior margins and potential underdosing of the prostatic peripheral zone (29, 30) (**Figure 1**). These tight posterior margins (< 1-2 mm) may only be feasible with fastidious motion management (31).

Improvements in target volume coverage as a result of spacer placement can be difficult to identify using standard instruments for plan evaluation in the clinical setting (32). Traditional dose-volume histogram (DVH) analysis lacks any positional data (33), and consequently it is an imprecise instrument to identify risk of recurrence when small portions of the prostate are underdosed. For example, the peripheral zone is the most common site of origin with the prostate gland for cancer development (34), and inadequate dose in small portions of this volume have been associated with increased risk of recurrence (35).

## Rationale for Utilizing Hydrogel Rectal Spacers for Prostate SBRT

**Figure 2:**
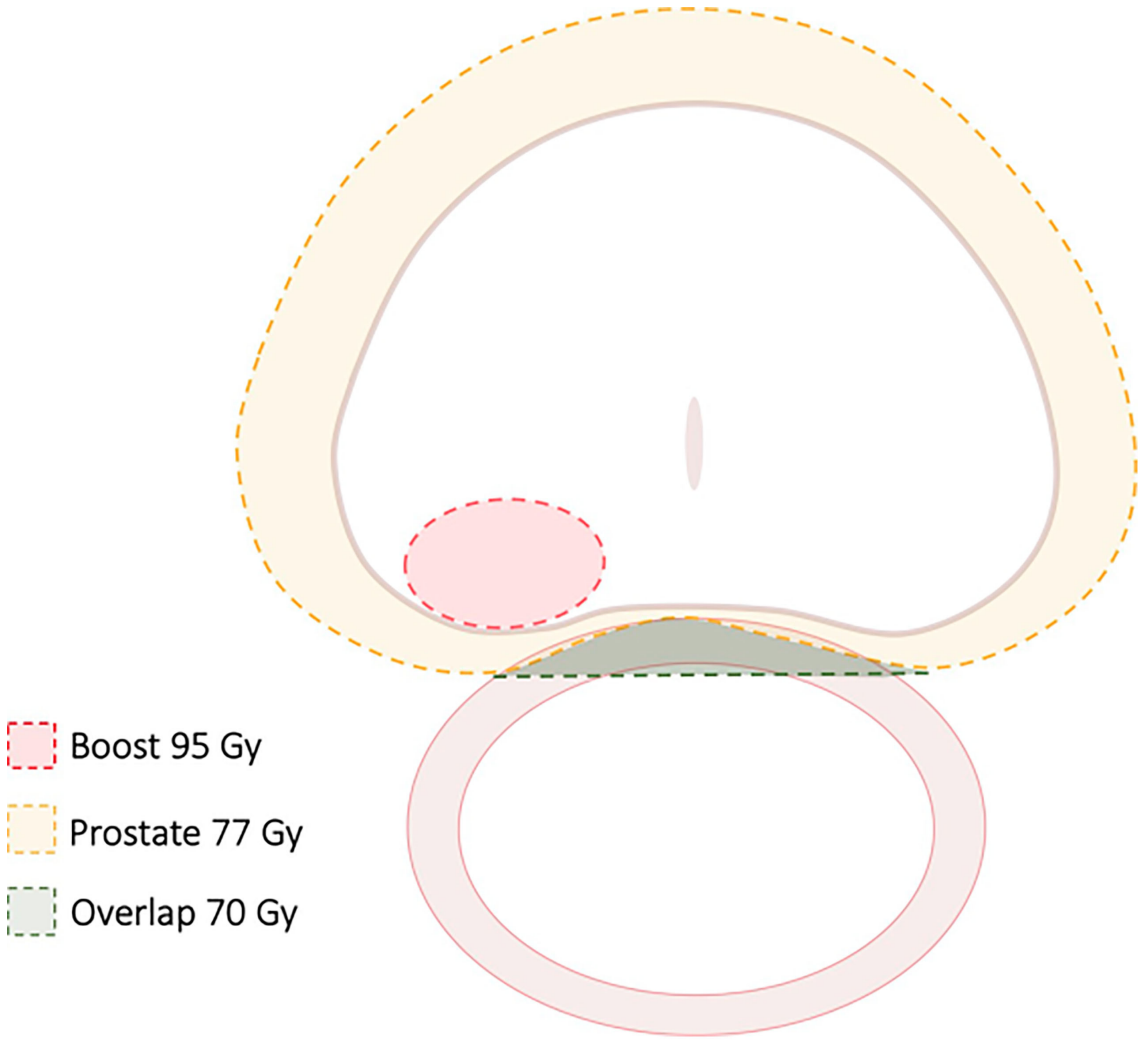
Treatment Guidelines for FLAME study. In this randomized study of focal dose escalation, in the experimental arm patients received 77 Gy to the PTV (70 Gy where there was overlap with the rectum) and 95 Gy to the MRI-defined GTV in 35 fractions using a simultaneous integrated boost.

**Figure 3:**
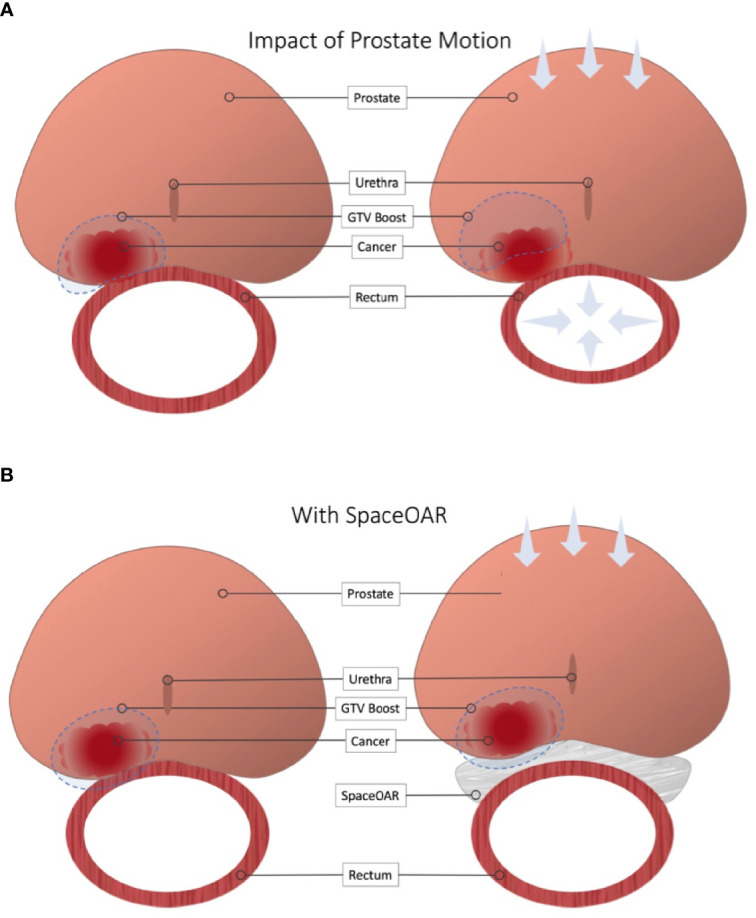
Impact of Rectal Spacer in Focal Dose Escalation. **(A)** Close proximity to the rectal wall can necessitate compromises in order to meet OAR constraints. Furthermore, minimal or omitted boost margins mean slight changes in local anatomy can cause a geographic miss. **(B)** Placement of a rectal spacer allows for greater boost margins and safer dose escalation.

**Figure 4:**
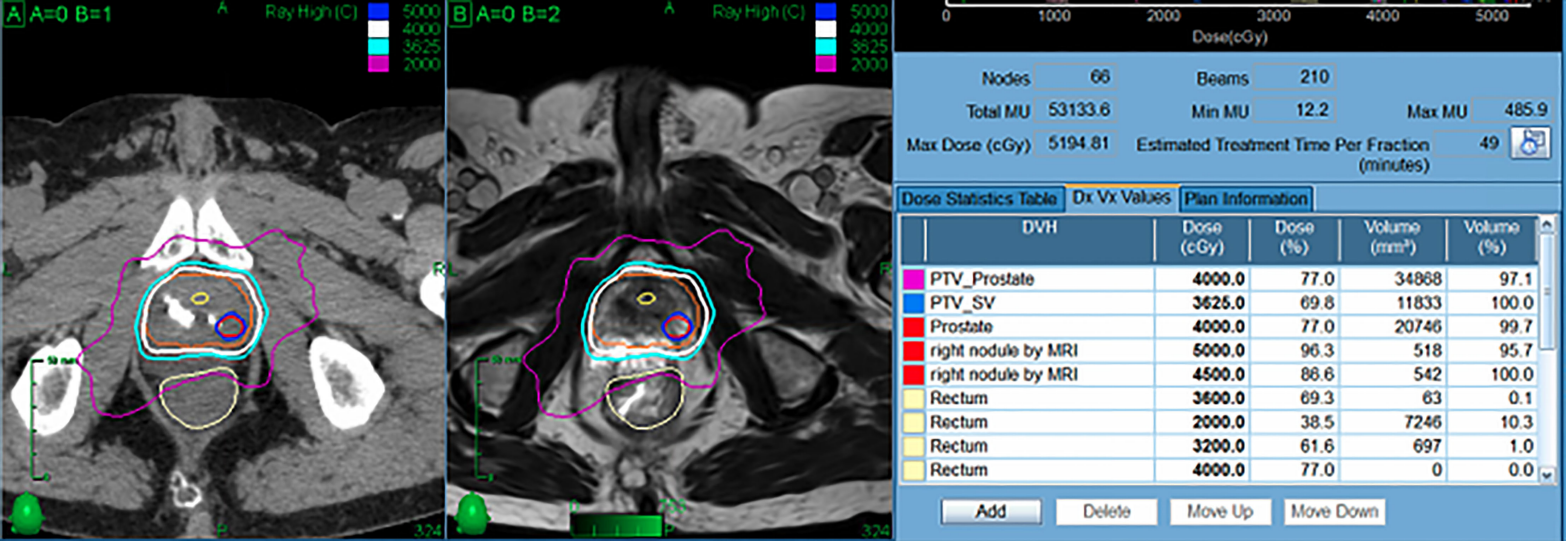
Representative SBRT patient treated with focal dose escalation and hydrogel rectal spacer. The patient received 40 Gy in 5 fractions to the prostate with an integrated boost to 50 Gy while maintaining excellent OAR dosimetry.

A recent randomized trial (FLAME) examined patients treated with conventional radiation (77 Gy in 2.2 Gy fractions) while using a simultaneous integrated boost (SIB) to deliver up to 95 Gy to an MRI-defined visible intraprostatic lesion (36) (**Figure 2**). There was a seven percent absolute improvement in biochemical disease-free survival (bDFS) at 5 years, without statistically significant changes in late toxicity and health-related QoL. However, standard dose constraints in this study were strictly enforced, and the mean dose delivered to the MRI-defined GTV (without PTV expansion) was lower at 91.9 Gy (37) (**Figure 3**). Given that higher GTV dose predicted increased 7-year biochemical disease-free survival (bDFS), it is reasonable to hypothesize that this benefit might have been greater with more comprehensive target coverage (**Figure 3**). In a follow-up phase II trial (hypo-FLAME), patients received SBRT (35 Gy in 5 fractions) with an SIB up to 50 Gy (38). While this approach was also well tolerated with low rates of late gastrointestinal and genitourinary toxicity, MRI-defined lesion coverage was even more difficult to achieve, with a median D99% of 40.3 Gy in this cohort. These doses may be more readily achievable with a well-placed rectal spacer (**Figure 4**).

In patients treated with SBRT, the use of a rectal spacer has demonstrated improvements in rectoprostatic separation (1.1 cm mean displacement), reduction of moderate and high rectal doses when tight PTV margins are utilized, and improvement of target volume coverage (39). Further, this displacement may lead to clinically reduced GI toxicity (40). Additionally, results from a recent prostate SBRT Phase I dose escalation study showed improved rates of pathological tumor clearance observed with higher doses (41). Preliminary data from the same group suggest that dose escalation may be even more important in unfavorable risk patients with higher tumor burdens (42). These data are supported by a recent tumor control probability (TCP) analysis that demonstrated higher doses are required to achieve a TCP of 95% in high risk patients (43). Early data from high risk patients suggest that 40 Gy in 5 fractions may improve bDFS at the cost of increased rectal toxicity when SBRT is performed without the use of a rectal spacer (44). A recent multi-institutional study of dose-escalated SBRT to 45 Gy in 5 fractions did in fact show >80% reduction in visualized rectal ulceration compared to previously observed rates in a similar patient cohort with the use of hydrogel Space OAR (45).

These studies indicate that dose escalation can produce meaningful clinical benefits for prostate cancer patients, albeit with an associated increased risk of severe long-term toxicity. While cautious planning can effectively mitigate these risks, this strategy requires sacrificing target coverage objectives and potentially abrogating or blunting the positive effects of escalation. One such approach is to deliver a moderate level of dose escalation to the entire prostate with ablative SBRT doses to suspected regions of highest-grade disease. Investigators on the CK-DESPOT study deliver 40 Gy in 5 fractions to the entire prostate while delivering 45-50 Gy to PI-RADS 4-5 nodules (**Figure 4**) (46). At a median follow-up of 18 months, no grade >2 GI toxicity has been recorded. (O. Obayomi-Davies, Personal Communication, January 2022)

Moreover, even patients undergoing conventionally-dosed radiotherapy for localized prostate cancer benefit from placement of a hydrogel rectal spacer. As discussed previously, rectal spacers reduce GI toxicity and maintain bowel quality of life following standard dose IMRT, and these benefits may be markedly more pronounced in patients at increased risk for high grade toxicity including those with inflammatory bowel disease on anticoagulants, though institutional reports suggest acceptable toxicity in these populations with standard dose SBRT (47–49). Taken together, these data strongly suggest a clear use for hydrogel spacers to decrease toxicity, improve target coverage, and achieve safer, more comprehensive dose escalation.

## Rationale for Utilizing Iodinated Hydrogel Rectal Spacers for Prostate SBRT

One major downside to the first generation rectal spacer is the similar radiodensities of the hydrogel and soft tissues such as the prostate and rectum. Consequently, these rectal spacers are difficult to visualize on CT scans, which can make accurate contouring challenging. For optimal delineation, a treatment planning MRI is required, but image registration and fusion is inherently inaccurate, leading to uncertainties in the gel interface with the prostate and rectum. This may lead to inaccuracies in target and OAR dose calculations. The importance of these inaccuracies is exacerbated when the prescription dose is escalated. Iodinated rectal spacers are readily visible on CT scan without altering the MRI appearance, thereby improving delineation of target volumes and OARs, which in turn helps ensure accurate dose delivery.

## Contraindications to Hydrogel Rectal Spacer Placement

**Figure 5:**
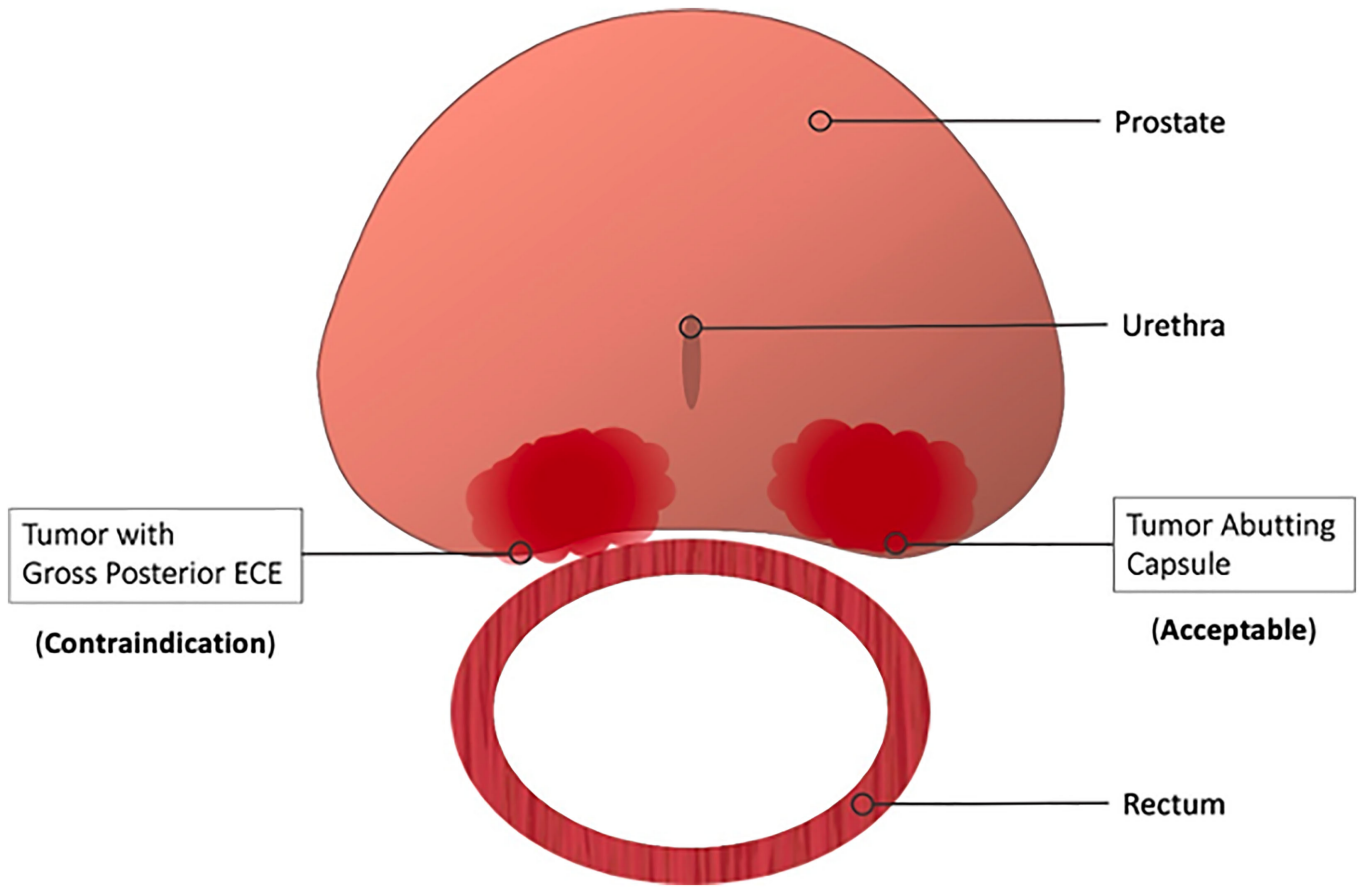
Contraindications to Hydrogel Rectal Spacer Placement. Most practitioners consider gross or radiographic posterior ECE a contraindication to spacer placement. Contrarily, spacer placement is acceptable in those patients with capsular abutment only.

Per the manufacturer’s labeling, there are no explicit contraindications to hydrogel rectal spacer placement for either the SpaceOAR or the SpaceOAR Vue hydrogels. While this may be accurate from a safety perspective, debate rages within the radiation oncology community as to whether any oncologic contraindications to treatment exist. Some practitioners advocate caution in patients with radiographic evidence of posterior extracapsular extension (ECE), while most do not consider posterior capsule abutment a contraindication (**Figure 5**). Due to these concerns, patients with more than 50% core positivity or radiographic ECE were excluded from the phase III rectal spacer trial (18). Theoretically, placing a rectal spacer in this situation might inadvertently “push” prostate cancer cells towards the rectum and beyond the area of high-dose radiotherapy, leading to higher rates of local failure.

Another potential concern with the SpaceOAR Vue system is the presence of iodine within the hydrogel and its safety in patients with an allergy to iodinated-contrast media. Per the manufacturer’s labeling “the use of this product in patients with documented iodine sensitivities or allergies has not been extensively studied. The risks and benefits of the decision to use in patients with a documented iodine allergy should be carefully considered on a case-by-case basis” (50). However, as the iodine molecules are covalently bonded to the hydrogel preventing their release into systemic circulation, the spacer should theoretically be well tolerated even in those patients with a true contrast allergy (21).

## Hydrogel Rectal Spacer Placement Procedure

Accurate placement is critical to maximize the benefits afforded by the hydrogel rectal spacer, and this is especially true when employing dose escalated regimens (51). Typically placement is performed at the same time as fiducial marker placement (or brachytherapy), adding minimal procedural time (52). The patient is positioned in the usual dorsal lithotomy position as he would be for a transperineal biopsy, fiducial placement, or brachytherapy procedure. Choice of anesthesia is at the discretion of the treating physician and anesthesiology team, but successful spacer placement has been performed under local anesthesia, light sedation, and general anesthesia. As the procedure is short and involves only a single transperineal needle, patients who undergo placement with only local anesthesia typically report minimal pain or discomfort (53). Similar to transperineal biopsy or brachytherapy, the risk of infection is much lower than transrectal procedures; while many centers employ prophylactic antibiotics others do not (54).

Once the patient is positioned, the ultrasound probe is placed within the rectum to visualize the prostate. Other procedures, such as fiducial placement or brachytherapy, should be performed prior to spacer placement as the gel can interfere with visualization of the gland on ultrasound. Placement of the needle in the correct plane and adequate hydrodissection are critical components of the procedure to ensure a high-quality spacer implant for optimal dosimetry (55). An 18G needle is placed bevel-down in the midline perineum approximately 2 cm above the TRUS probe angled slightly (~15 degrees) posteriorly. The sagittal viewing plane is used as the needle is advanced to the mid-gland in the space anterior to the rectum and posterior to the rectoprostatic fascia. The axial viewing plane should be utilized to confirm midline position of the needle, with slight needle movements to ensure the needle has not been introduced into the anterior rectal wall. A small “puff” of saline is then employed to confirm placement prior to full hydrodissection of the space with approximately 10 mL of saline. Prior to proceeding with hydrogel placement, a small amount of fluid should be aspirated to ensure the needle is not placed intravascularly.

The saline syringe is then removed, and hydrogel applicator attached to the needle. The hydrogel is then slowly injected into the hydrodissected space. For placement of the original SpaceOAR, the hydrogel is injected over 10 seconds, while the radio-opaque SpaceOAR Vue is injected over approximately 20 seconds. Once injection is started, it is critical that it be done in a continuous, smooth motion without stops to prevent polymerization and clogging within the needle. The needle is then removed, completing the procedure.

## Procedure-Related Risks of Rectal Spacer Placement

In general, the procedure is well tolerated with limited risk of adverse effects. Some patients have reported self-limited discomfort and rectal tenesmus following the procedure, though this appears to be relatively uncommon (18). Though cases of inadvertent injection of hydrogel into the rectal wall or bladder have been reported, the majority of these resolve with conservative management and time, which allows the hyrdrogel to slowly resorb (56, 57). Careful review of treatment planning imaging is required prior to radiotherapy to ensure appropriate spacer position.

Nonetheless, some practitioners do advocate caution before routine adoption of a hydrogel rectal spacer in all prostate cancer patients slated for radiotherapy (58). Common counter-arguments include a failure in the phase III trial to meet the primary safety endpoint (grade 1+ rectal or procedural adverse events in the first 6 months: 34·2% *vs* 31·5%, p=0.7), although secondary analyses demonstrated significant benefits in both practitioner-graded toxicities and patient-reported outcomes. Additionally, a small study of the FDA Manufacturer and User Facility Device (MAUDE) database reported severe complications in a small number of patients, 11 of whom required surgical intervention, following hydrogel rectal spacer placement (59). These complications included perineal abscess requiring drainage, rectourethral fistula, proctitis requiring colostomy, and severe urosepsis necessitating ICU level care. Two patients died following spacer placement, although in one case the cause of death was uncertain and in the other it was unrelated to the rectal spacer. Ultimately, the quality of spacer placement, benefit to the patient and potential risks are dependent on the individual provider’s ability and experience, underscoring the importance of proper training and certification for providers who wish to place rectal spacers in their patients. Finally, controversy and uncertainty persist regarding the cost-effectiveness of the procedure (60).

## Assessment of Spacer Placement and Planning Considerations

Given the limitations of trans-rectal ultrasound image quality, optimal assessment of rectal spacer placement is typically performed at the time of radiotherapy simulation. In addition to typical CT-based simulation, a dedicated treatment planning MR scan is preferred for optimal evaluation, although the advent of an iodinated, radio-opaque spacer has made treatment possible for those patients with a contraindication (21).

In the phase III spacer trial, the mean peri-rectal distance was 1.6 mm prior to placement and 12.6 mm following hydrogel application, consistent with other institutional and dosimetric studies (18). In this patient population, a secondary, *post-hoc* semi-qualitative analysis of hydrogel symmetry demonstrated that approximately 50% of patients had fully symmetrical spacer placement all levels assessed, though only 32% of patients had hydrogel present at both the base and the apex (61). Nonetheless, a 25% reduction in the rectal volume receiving at least 70 Gy (V70) with the addition of a rectal spacer was achieved in greater than 97% of patients, suggesting that the overwhelming majority of patients experience a clinical benefit even in the face of suboptimal spacer placement. Multiple other non-randomized studies have recapitulated similar results in large patient populations, and there is some evidence to suggest a learning curve effect with improvements in placement quality over time (62), consistent with similar trends observed in patients undergoing brachytherapy (63).

Concerns have arisen regarding the clinical implications of rectal wall infiltration (RWI) as identified on treatment planning MRIs. Six percent of patients on the aforementioned phase III study were noted to have RWI of the hydrogel, though more than half of these cases consisted of “small, discrete areas” of infiltration, while only a single patient was noted to have radiographic involvement of more than 25% of the rectal wall circumference (61). Fortunately, there was no identifiable increase in toxicity in patients with RWI, and the one patient with substantial RWI experienced no procedural, acute, or late toxicity. Nonetheless, RWI should be treated with a high degree of caution given the possibility of catastrophic toxicity if it is not identified prior to definitive treatment. In one case report of a patient undergoing dose-escalated SBRT (45 Gy in 5 fractions) with a rectal spacer, RWI was not identified during treatment planning, and the patient ultimately required abdomino-perineal resection (APR), cystoprostatectomy, and ileal conduit placement secondary to complications from a large recto-urethral fistula (51). In retrospect, hydrogel was identified within the submucosa of the rectum, secondary to delamination and discontinuity of the muscularis propria. The authors of this report suggest careful evaluation of planning MRI scans for RWI and referral for endoscopic evaluation in cases of concern, with a low threshold to delay treatment until spacer resorption if any abnormalities are noted. Thankfully, while this case highlights the need for careful radiographic assessment of spacer placement, it also represents an extremely rare outlier from an otherwise safe procedure.

**Figure 6:**
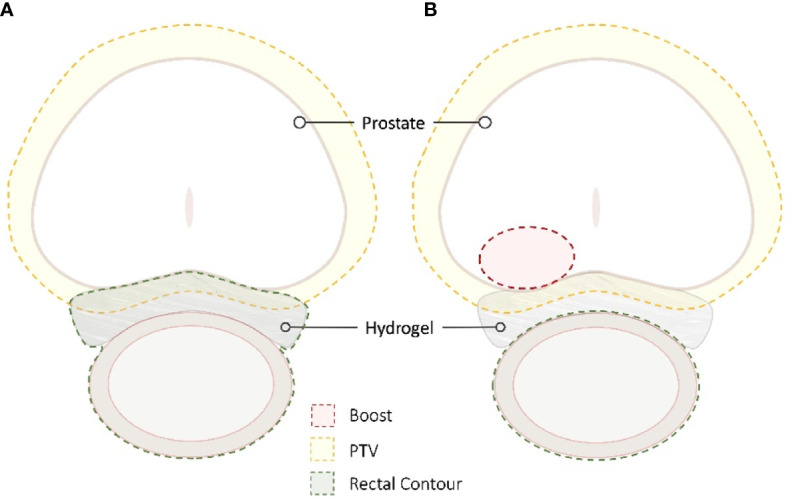
SBRT Treatment Planning. The radiation sensitivity of the rectum in patients with inflammatory bowel disease is unknown; the goal is to decrease the rectal dose to as low as reasonably achievable (ALARA). For many patients, the hydrogel is incorporated into the rectal contour to maximize rectal spacing **(A)**. In other patients, the spacer is not incorporated to allow for dose escalation while maintaining strict rectal dose constraints **(B)**.

The optimal rectal dose constraints for patients with a rectal spacer undergoing conventionally fractionated radiotherapy, moderately hypofractionated radiotherapy, or SBRT are currently unknown. At a minimum, typical dose constraints used in non-spacer patients should be easily achievable with placement of a spacer (21), and should ideally allow for safer dose escalation (64). One frequently employed strategy to aggressively manage rectal dose is to contour the spacer itself as part of the rectal contour, although retrospective data suggest that this approach may not yield optimal treatment plans (65) (**Figure 6**).

## Conclusions and Future Directions

With the results of the recently published HYPO-RT-PC trial demonstrating excellent outcomes for prostate cancer patients treated with extreme hypofractionation as well as the disincentive to longer treatment courses predicted with implementation of the forthcoming radiation oncology payment model, utilization of SBRT is expected to increase dramatically in the coming decade (66–68). However, despite its recent adoption as an acceptable front-line treatment in the National Cancer Consensus Network (NCCN) guidelines (69), optimal dose constraints remain nebulous, especially as dose escalation becomes more widespread. Yet even with this uncertainty, placement of a hydrogel rectal spacer produces dosimetric improvements as well as clinically significant decreases in toxicity that may make it indispensable in treatment of prostate cancer patients, particularly those with high-risk disease.

Multiple forthcoming trials seek to refine dose-escalated and hypofractionated radiation schema, and the use of a hydrogel rectal spacer will be essential in many of these studies. For example, the ongoing SABRE (Effectiveness of the SpaceOAR Vue System in Subjects with Prostate Cancer being Treated with Stereotactic Body Radiotherapy) is a multi-center, prospective, randomized study which will evaluate the role of the SpaceOAR Vue in patients with intermediate risk prostate cancer (70). Patients on this study will receive dose-escalated SBRT (40 Gy in 5 fractions) with the primary outcome measure of a reduction in late GI toxicity (Grade 2+ between 3- and 24-months post-treatment).

In summary, placement of a hydrogel rectal spacer is a low-risk procedure that produces meaningful clinical benefits for patients undergoing definitive radiotherapy for localized prostate cancer. Dosimetric improvements are noted in the vast majority of cases, even when rectal spacer placement is suboptimal, though careful assessment of hydrogel placement is required for each patient. Complications associated with spacer placement; especially severe adverse events are rare. Ongoing studies will help to clarify the potential benefits in patients undergoing dose-escalated and hypofractionated regimens.

## Author Contributions

MR wrote the initial draft of the manuscript. MCr and OO-D created the figures. All authors participated in editing and review of the manuscript. All authors contributed to the article and approved the submitted version.

## Conflict of Interest

SC serves as a consultant for Accuray (Sunnyvale, CA) and Boston Scientific (Marlborough, MA).

The remaining authors declare that the research was conducted in the absence of any commercial or financial relationships that could be construed as a potential conflict of interest.

## Publisher’s Note

All claims expressed in this article are solely those of the authors and do not necessarily represent those of their affiliated organizations, or those of the publisher, the editors and the reviewers. Any product that may be evaluated in this article, or claim that may be made by its manufacturer, is not guaranteed or endorsed by the publisher.

## References

[1] Siegel RL Miller KD Fuchs HE Jemal A. Cancer Statistics, 2021. CA Cancer J Clin (2021) 71(1):7–33. doi: 10.3322/caac.21654 33433946

[2] Kalbasi A Li J Berman AT Swisher-McClure S Smaldone M Uzzo RG. Dose-Escalated Irradiation and Overall Survival in Men With Nonmetastatic Prostate Cancer. JAMA Oncol (2015) 1(7):897–906. doi: 10.1001/jamaoncol.2015.2316 26181727

[3] Pinkawa M Holy R Piroth MD Fischedick K Schaar S Székely-Orbán D. Consequential Late Effects After Radiotherapy for Prostate Cancer - A Prospective Longitudinal Quality of Life Study. Radiat Oncol (2010) 5(1):27. doi: 10.1186/1748-717X-5-27 20377874 PMC2857853

[4] Hamdy FC Donovan JL Lane JA Mason M Metcalfe C Holding P. 10-Year Outcomes After Monitoring, Surgery, or Radiotherapy for Localized Prostate Cancer. N Engl J Med (2016) 375(15):1415–24. doi: 10.1056/NEJMoa1606220 27626136

[5] Donovan JL Hamdy FC Lane JA Mason M Metcalfe C Walsh E. Patient-Reported Outcomes After Monitoring, Surgery, or Radiotherapy for Prostate Cancer. N Engl J Med (2016) 375(15):1425–37. doi: 10.1056/NEJMoa1606221 PMC5134995 27626365

[6] Kim S Shen S Moore DF Shih W Lin Y Li H. Late Gastrointestinal Toxicities Following Radiation Therapy for Prostate Cancer. Eur Urol (2011) 60(5):908–16. doi: 10.1016/j.eururo.2011.05.052 PMC3185133 21684064

[7] Michalski JM Yan Y Watkins-Bruner D Bosch WR Winter K Galvin JM. Preliminary Toxicity Analysis of 3-Dimensional Conformal Radiation Therapy Versus Intensity Modulated Radiation Therapy on the High-Dose Arm of the Radiation Therapy Oncology Group 0126 Prostate Cancer Trial. Int J Radiat Oncol (2013) 87(5):932–8. doi: 10.1016/j.ijrobp.2013.07.041 PMC3840044 24113055

[8] Sheets NC Goldin GH Meyer A-M Wu Y Chang Y Stürmer T. Intensity-Modulated Radiation Therapy, Proton Therapy, or Conformal Radiation Therapy and Morbidity and Disease Control in Localized Prostate Cancer. JAMA (2012) 307(15):1611–20. doi: 10.1001/jama.2012.460 PMC3702170 22511689

[9] Teh BS McGary JE Dong L Mai W-Y Carpenter LS Lu HH. The Use of Rectal Balloon During the Delivery of Intensity Modulated Radiotherapy (IMRT) for Prostate Cancer: More Than Just a Prostate Gland Immobilization Device? Cancer J Sudbury Mass (2002) 8(6):476–83. doi: 10.1097/00130404-200211000-00012 12500857

[10] Villers A Mcneal JE Freiha FS Boccon-Gibod L Stamey TA. Invasion Of Denonvilliers’ Fascia in Radical Prostatectomy Specimens. J Urol (1993) 149(4):793–8. doi: 10.1016/S0022-5347(17)36209-2 8455242

[11] Rosenberg GS Basralian KG. Active Hydrodissection Might Optimize Cryosurgical Ablation of the Prostate. Urology (2010) 76(4):988–91. doi: 10.1016/j.urology.2009.12.081 20537688

[12] Melchert C Gez E Bohlen G Scarzello G Koziol I Anscher M. Interstitial Biodegradable Balloon for Reduced Rectal Dose During Prostate Radiotherapy: Results of a Virtual Planning Investigation Based on the Pre- and Post-Implant Imaging Data of an International Multicenter Study. Radiother Oncol (2013) 106(2):210–4. doi: 10.1016/j.radonc.2013.01.007 23484879

[13] Chapet O Udrescu C Devonec M Tanguy R Sotton M-P Enachescu C. Prostate Hypofractionated Radiation Therapy: Injection of Hyaluronic Acid to Better Preserve The Rectal Wall. Int J Radiat Oncol (2013) 86(1):72–6. doi: 10.1016/j.ijrobp.2012.11.027 23290444

[14] Susil RC McNutt TR DeWeese TL Song D. Effects of Prostate-Rectum Separation on Rectal Dose From External Beam Radiotherapy. Int J Radiat Oncol (2010) 76(4):1251–8. doi: 10.1016/j.ijrobp.2009.07.1679 PMC3115781 19939577

[15] Garasic JM Marin L Anderson RD. Acute Evaluation of the Mynx Vascular Closure Device During Arterial Re-Puncture in an Ovine Model. J Invasive Cardiol (2009) 21(6):283–5. 19494406

[16] Cosgrove GR Delashaw JB Grotenhuis JA Tew JM van Loveren H Spetzler RF. Safety and Efficacy of a Novel Polyethylene Glycol Hydrogel Sealant for Watertight Dural Repair. J Neurosurg (2007) 106(1):52–8. doi: 10.3171/jns.2007.106.1.52 17236487

[17] Bertolaccini L Lybéris P Manno E. Lung Sealant and Morbidity After Pleural Decortication: A Prospective Randomized, Blinded Study. J Cardiothorac Surg (2010) 5(1):45. doi: 10.1186/1749-8090-5-45 20509919 PMC2907570

[18] Mariados N Sylvester J Shah D Karsh L Hudes R Beyer D. Hydrogel Spacer Prospective Multicenter Randomized Controlled Pivotal Trial: Dosimetric and Clinical Effects of Perirectal Spacer Application in Men Undergoing Prostate Image Guided Intensity Modulated Radiation Therapy. Int J Radiat Oncol (2015) 92(5):971–7. doi: 10.1016/j.ijrobp.2015.04.030 26054865

[19] Augmenix Announces FDA Clearance of SpaceOAR® System [Internet] (2015). Available at: https://www.businesswire.com/news/home/20150402005778/en/Augmenix-Announces-FDA-Clearance-of-SpaceOAR%C2%AE-System (Accessed cited 2021 Oct 26).

[20] Boston Scientific Closes Acquisition of Augmenix, Inc. Boston Scientific. Available at: https://news.bostonscientific.com/2018-10-16-Boston-Scientific-Closes-Acquisition-of-Augmenix-Inc (Accessed cited 2021 Oct 26).

[21] Conroy D Becht K Forsthoefel M Pepin AN Lei S Rashid A. Utilization of Iodinated SpaceOAR Vue™ During Robotic Prostate Stereotactic Body Radiation Therapy (SBRT) to Identify the Rectal–Prostate Interface and Spare the Rectum: A Case Report. Front Oncol (2021) 10:607698. doi: 10.3389/fonc.2020.607698 33489918 PMC7817609

[22] Hamstra DA Mariados N Sylvester J Shah D Karsh L Hudes R. Continued Benefit to Rectal Separation for Prostate Radiation Therapy: Final Results of a Phase III Trial. Int J Radiat Oncol (2017) 97(5):976–85. doi: 10.1016/j.ijrobp.2016.12.024 28209443

[23] Hamstra DA Mariados N Sylvester J Shah D Gross E Hudes R. Sexual Quality of Life Following Prostate Intensity Modulated Radiation Therapy (IMRT) With a Rectal/Prostate Spacer: Secondary Analysis of a Phase 3 Trial. Pract Radiat Oncol (2018) 8(1):e7–15. doi: 10.1016/j.prro.2017.07.008 28951089

[24] Quinn TJ Daignault-Newton S Bosch W Mariados N Sylvester J Shah D. Who Benefits From a Prostate Rectal Spacer? Secondary Analysis of a Phase III Trial. Pract Radiat Oncol (2020) 10(3):186–94. doi: 10.1016/j.prro.2019.12.011 31978591

[25] Meier RM Bloch DA Cotrutz C Beckman AC Henning GT Woodhouse SA. Multi-Center Trial of Stereotactic Body Radiotherapy for Low- and Intermediate-Risk Prostate Cancer: Survival and Toxicity Endpoints(2018). Available at: https://www.redjournal.org/article/S0360-3016(18)30891-5/fulltext (Accessed cited 2018 Jun 9). 10.1016/j.ijrobp.2018.05.040 30191864

[26] Kim DWN Cho LC Straka C Christie A Lotan Y Pistenmaa D. Predictors of Rectal Tolerance Observed in a Dose-Escalated Phase 1-2 Trial of Stereotactic Body Radiation Therapy for Prostate Cancer. Int J Radiat Oncol Biol Phys (2014) 89(3):509–17. doi: 10.1016/j.ijrobp.2014.03.012 24929162

[27] Kim DWN Straka C Cho LC Timmerman RD. Stereotactic Body Radiation Therapy for Prostate Cancer: Review of Experience of a Multicenter Phase I/II Dose-Escalation Study. Front Oncol (2014) 4:319. doi: 10.3389/fonc.2014.00319 25505731 PMC4245005

[28] Hannan R Tumati V Xie X-J Cho LC Kavanagh BD Brindle J. Stereotactic Body Radiation Therapy for Low and Intermediate Risk Prostate Cancer—Results From a Multi-Institutional Clinical Trial. Eur J Cancer (2016) 59:142–51. doi: 10.1016/j.ejca.2016.02.014 27035363

[29] Ju AW Wang H Oermann EK Sherer BA Uhm S Chen VJ. Hypofractionated Stereotactic Body Radiation Therapy as Monotherapy for Intermediate-Risk Prostate Cancer. Radiat Oncol Lond Engl (2013) 8:30. doi: 10.1186/1748-717X-8-30 PMC3570380 23369294

[30] Joh DY Chen LN Porter G Bhagat A Sood S Kim JS. Proctitis Following Stereotactic Body Radiation Therapy for Prostate Cancer. Radiat Oncol (2014) 9(1):277. doi: 10.1186/s13014-014-0277-4 25497602 PMC4272823

[31] Xie Y Djajaputra D King CR Hossain S Ma L Xing L. Intrafractional Motion of the Prostate During Hypofractionated Radiotherapy. Int J Radiat Oncol Biol Phys (2008) 72(1):236–46. doi: 10.1016/j.ijrobp.2008.04.051 PMC2725181 18722274

[32] Ruggieri R Naccarato S Stavrev P Stavreva N Fersino S Giaj Levra N. Volumetric-Modulated Arc Stereotactic Body Radiotherapy for Prostate Cancer: Dosimetric Impact of an Increased Near-Maximum Target Dose and of a Rectal Spacer. Br J Radiol (2015) 88(1054):20140736. doi: 10.1259/bjr.20140736 26235142 PMC4738106

[33] Drzymala RE Mohan R Brewster L Chu J Goitein M Harms W. Dose-Volume Histograms. Int J Radiat Oncol Biol Phys (1991) 21(1):71–8. doi: 10.1016/0360-3016(91)90168-4 2032898

[34] McNeal JE Redwine EA Freiha FS Stamey TA. Zonal Distribution of Prostatic Adenocarcinoma. Correlation With Histologic Pattern and Direction of Spread. Am J Surg Pathol (1988) 12(12):897–906. doi: 10.1097/00000478-198812000-00001 3202246

[35] Murakami Y Soyano T Kozuka T Ushijima M Koizumi Y Miyauchi H. Dose-Based Radiomic Analysis (Dosiomics) for Intensity Modulated Radiation Therapy in Patients With Prostate Cancer: Correlation Between Planned Dose Distribution and Biochemical Failure. Int J Radiat Oncol Biol Phys (2022) 112(1):247–59. doi: 10.1016/j.ijrobp.2021.07.1714 34706278

[36] Kerkmeijer LGW Groen VH Pos FJ Haustermans K Monninkhof EM Smeenk RJ. Focal Boost to the Intraprostatic Tumor in External Beam Radiotherapy for Patients With Localized Prostate Cancer: Results From the FLAME Randomized Phase III Trial. J Clin Oncol Off J Am Soc Clin Oncol (2021) 39(7):787–96. doi: 10.1200/JCO.20.02873 33471548

[37] Monninkhof EM van Loon JWL van Vulpen M Kerkmeijer LGW Pos FJ Haustermans K. Standard Whole Prostate Gland Radiotherapy With and Without Lesion Boost in Prostate Cancer: Toxicity in the FLAME Randomized Controlled Trial. Radiother Oncol (2018) 127(1):74–80. doi: 10.1016/j.radonc.2017.12.022 29336835

[38] Draulans C van der Heide UA Haustermans K Pos FJ van der Voort van Zyp J De Boer H. Primary Endpoint Analysis of the Multicentre Phase II Hypo-FLAME Trial for Intermediate and High Risk Prostate Cancer. Radiother Oncol J Eur Soc Ther Radiol Oncol (2020) 147:92–8. doi: 10.1016/j.radonc.2020.03.015 32247206

[39] Kataria S Hong RL McRae D Cernica G Foust M Nasr NM. The Rectal Dosimetric Effects of Perirectal Hydrogel Spacers in Men Undergoing Prostate Stereotactic Body Radiation Therapy (SBRT). Int J Radiat Oncol Biol Phys (2017) 99(2):E676. doi: 10.1016/j.ijrobp.2017.06.2233

[40] Payne HA Pinkawa M Peedell C Bhattacharyya SK Woodward E Miller LE. SpaceOAR Hydrogel Spacer Injection Prior to Stereotactic Body Radiation Therapy for Men With Localized Prostate Cancer. Med (Baltimore) (2021) 100(49):e28111. doi: 10.1097/MD.0000000000028111 PMC8663810 34889268

[41] Zelefsky MJ Kollmeier M McBride S Varghese M Mychalczak B Gewanter R. Five-Year Outcomes of a Phase 1 Dose-Escalation Study Using Stereotactic Body Radiosurgery for Patients With Low-Risk and Intermediate-Risk Prostate Cancer. Int J Radiat Oncol Biol Phys (2019) 104(1):42–9. doi: 10.1016/j.ijrobp.2018.12.045 PMC7525798 30611838

[42] Zelefsky MJ Pinitpatcharalert A Goldman DA Kollmeier MA Hopkins M McBride S. Higher SBRT Dose Levels for Localized Prostate Cancer Are Associated With Improved Post-Treatment Biopsy Outcomes. Int J Radiat Oncol Biol Phys (2020) 108(3):e882. doi: 10.1016/j.ijrobp.2020.07.476

[43] Royce TJ Mavroidis P Wang K Falchook AD Sheets NC Fuller DB. Tumor Control Probability Modeling and Systematic Review of the Literature of Stereotactic Body Radiation Therapy for Prostate Cancer. Int J Radiat Oncol Biol Phys (2021) 110(1):227–36. doi: 10.1016/j.ijrobp.2020.08.014 PMC9445430 32900561

[44] van Dams R Jiang NY Fuller DB Loblaw A Jiang T Katz AJ. Stereotactic Body Radiotherapy for High-Risk Localized Carcinoma of the Prostate (SHARP) Consortium: Analysis of 344 Prospectively Treated Patients. Int J Radiat Oncol (2021) 110(3):731–7. doi: 10.1016/j.ijrobp.2021.01.016 PMC8956505 33493615

[45] Folkert MR Zelefsky MJ Hannan R Desai NB Lotan Y Laine AM. A Multi-Institutional Phase 2 Trial of High-Dose SAbR for Prostate Cancer Using Rectal Spacer. Int J Radiat Oncol Biol Phys (2021) 111(1):101–9. doi: 10.1016/j.ijrobp.2021.03.025 33753140

[46] Lanciano R. CyberKnife Dose Escalation for Unfavorable and High-Risk Prostate Cancer (2021). clinicaltrials.gov. Available at: https://clinicaltrials.gov/ct2/show/NCT03822494 (Accessed cited 2022 Jan 20). Report No.: NCT03822494.

[47] White EC Murphy JD Chang DT Koong AC. Low Toxicity in Inflammatory Bowel Disease Patients Treated With Abdominal and Pelvic Radiation Therapy. Am J Clin Oncol (2015) 38(6):564–9. doi: 10.1097/COC.0000000000000010 24401668

[48] Lischalk JW Blacksburg S Mendez C Repka M Sanchez A Carpenter T. Stereotactic Body Radiation Therapy for the Treatment of Localized Prostate Cancer in Men With Underlying Inflammatory Bowel Disease. Radiat Oncol Lond Engl (2021) 16(1):126. doi: 10.1186/s13014-021-01850-1 PMC8267228 34243797

[49] Pepin A Shah S Pernia M Lei S Ayoob M Danner M. Bleeding Risk Following Stereotactic Body Radiation Therapy for Localized Prostate Cancer in Men on Baseline Anticoagulant or Antiplatelet Therapy. Front Oncol (2021) 11:722852. doi: 10.3389/fonc.2021.722852 34604059 PMC8485025

[50] URO-855204-AA SpaceOAR VUE_Brief Summary.Pdf. Available at: https://www.bostonscientific.com/content/dam/bostonscientific/spaceoar/vue/URO-855204-AA%20SpaceOAR%20VUE_Brief%20Summary.pdf (Accessed cited 2021 Oct 26).

[51] McLaughlin MF Folkert MR Timmerman RD Hannan R Garant A Hudak SJ. Hydrogel Spacer Rectal Wall Infiltration Associated With Severe Rectal Injury and Related Complications After Dose Intensified Prostate Cancer Stereotactic Ablative Radiation Therapy(2021). Available at: https://www.advancesradonc.org/article/S2452-1094(21)00071-3/fulltext (Accessed cited 2021 Oct 20). 10.1016/j.adro.2021.100713 PMC8239444 34195499

[52] Hatiboglu G Pinkawa M Vallée J-P Hadaschik B Hohenfellner M. Application Technique: Placement of a Prostate–Rectum Spacer in Men Undergoing Prostate Radiation Therapy. BJU Int (2012) 110(11b):E647–52. doi: 10.1111/j.1464-410X.2012.11373.x 22788857

[53] Montoya J Gross E Karsh L. How I Do It: Hydrogel Spacer Placement in Men Scheduled to Undergo Prostate Radiotherapy. Can J Urol (2018) 25(2):9288–93. 29680009

[54] Pinkawa M. Spacer Application for Prostate Cancer Radiation Therapy. Future Oncol (2014) 10(5):851–64. doi: 10.2217/fon.13.223 24799065

[55] Fagundes M Rodrigues MA Olszewski S Khan F McKenzie C Gutierrez A. Expanding the Utilization of Rectal Spacer Hydrogel for Larger Prostate Glands (>80 Cc): Feasibility and Dosimetric Outcomes. Adv Radiat Oncol (2021) 6(3):100651. doi: 10.1016/j.adro.2021.100651 34195489 PMC8233470

[56] Uhl M Herfarth K Eble MJ Pinkawa M van Triest B Kalisvaart R. Absorbable Hydrogel Spacer Use in Men Undergoing Prostate Cancer Radiotherapy: 12 Month Toxicity and Proctoscopy Results of a Prospective Multicenter Phase II Trial. Radiat Oncol (2014) 9(1):96. doi: 10.1186/1748-717X-9-96 24758224 PMC4016630

[57] Uhl M van Triest B Eble MJ Weber DC Herfarth K De Weese TL. Low Rectal Toxicity After Dose Escalated IMRT Treatment of Prostate Cancer Using an Absorbable Hydrogel for Increasing and Maintaining Space Between the Rectum and Prostate: Results of a Multi-Institutional Phase II Trial. Radiother Oncol (2013) 106(2):215–9. doi: 10.1016/j.radonc.2012.11.009 23333011

[58] Hall WA Tree AC Dearnaley D Parker CC Prasad V Roach M. Considering Benefit and Risk Before Routinely Recommending SpaceOAR. Lancet Oncol (2021) 22(1):11–3. doi: 10.1016/S1470-2045(20)30639-2 PMC8882263 33387489

[59] Aminsharifi A Kotamarti S Silver D Schulman A. Major Complications and Adverse Events Related to the Injection of the SpaceOAR Hydrogel System Before Radiotherapy for Prostate Cancer: Review of the Manufacturer and User Facility Device Experience Database. J Endourol (2019) 33(10):868–71. doi: 10.1089/end.2019.0431 31452385

[60] Levy JF Khairnar R Louie AV Showalter TN Mullins CD Mishra MV. Evaluating the Cost-Effectiveness of Hydrogel Rectal Spacer in Prostate Cancer Radiation Therapy. Pract Radiat Oncol (2019) 9(2):e172–9. doi: 10.1016/j.prro.2018.10.003 30342180

[61] Fischer-Valuck BW Chundury A Gay H Bosch W Michalski J. Hydrogel Spacer Distribution Within the Perirectal Space in Patients Undergoing Radiotherapy for Prostate Cancer: Impact of Spacer Symmetry on Rectal Dose Reduction and the Clinical Consequences of Hydrogel Infiltration Into the Rectal Wall. Pract Radiat Oncol (2017) 7(3):195–202. doi: 10.1016/j.prro.2016.10.004 28089528

[62] Pinkawa M Klotz J Djukic V Schubert C Escobar-Corral N Caffaro M. Learning Curve in the Application of a Hydrogel Spacer to Protect the Rectal Wall During Radiotherapy of Localized Prostate Cancer. Urology (2013) 82(4):963–8. doi: 10.1016/j.urology.2013.07.014 24074991

[63] Chan EK Keyes M Pickles T Lapointe V Spadinger I McKenzie M. Decline in Acute Urinary Toxicity: A Long-Term Study in 2011 Patients With Prostate Brachytherapy Within a Provincial Institution. Brachytherapy (2014) 13(1):46–52. doi: 10.1016/j.brachy.2013.10.005 24210697

[64] M.D RL. CyberKnife Dose Escalation for Unfavorable and High-Risk Prostate Cancer (2021). clinicaltrials.gov. Available at: https://clinicaltrials.gov/ct2/show/NCT03822494 (Accessed cited 2021 Oct 24). Report No.: NCT03822494.

[65] Paetkau O Gagne IM Pai HH Lam J Goulart J Alexander A. Maximizing Rectal Dose Sparing With Hydrogel: A Retrospective Planning Study. J Appl Clin Med Phys (2019) 20(4):91–8. doi: 10.1002/acm2.12566 PMC6448161 30889318

[66] Widmark A Gunnlaugsson A Beckman L Thellenberg-Karlsson C Hoyer M Lagerlund M. Ultra-Hypofractionated Versus Conventionally Fractionated Radiotherapy for Prostate Cancer: 5-Year Outcomes of the HYPO-RT-PC Randomised, Non-Inferiority, Phase 3 Trial. Lancet (2019) 394(10196):385–95. doi: 10.1016/S0140-6736(19)31131-6 31227373

[67] Boyce-Fappiano D Ning MS Gjyshi O Mesko S Pasalic D Chang AJ. Payment Methodology for the Radiation Oncology Alternative Payment Model: Implications for Practices and Suggestions for Improvement. JCO Oncol Pract (2021) 17:OP.21.00200. doi: 10.1200/OP.21.00200 34097458

[68] Mahase SS D’Angelo D Kang J Hu JC Barbieri CE Nagar H. Trends in the Use of Stereotactic Body Radiotherapy for Treatment of Prostate Cancer in the United States. JAMA Netw Open (2020) 3(2):e1920471. doi: 10.1001/jamanetworkopen.2019.20471 32022878 PMC12068824

[69] Prostate.Pdf. Available at: https://www.nccn.org/professionals/physician_gls/pdf/prostate.pdf (Accessed cited 2021 Oct 26).

[70] Boston Scientific Corporation. Effectiveness of the SpaceOAR Vue System in Subjects With Prostate Cancer Being Treated With Stereotactic Body Radiotherapy (2021). clinicaltrials.gov. Available at: https://clinicaltrials.gov/ct2/show/NCT04905069 (Accessed cited 2021 Oct 24). Report No.: NCT04905069.

